# Protocol for the development of coarse-grained structures for macromolecular simulation using GROMACS

**DOI:** 10.1371/journal.pone.0288264

**Published:** 2023-08-03

**Authors:** Vidya Niranjan, Purushotham Rao, Akshay Uttarkar, Jitendra Kumar

**Affiliations:** 1 Department of Biotechnology, R V College of Engineering, Bengaluru, Karnataka, India; 2 Managing Director, Biotechnology Industry Research Assistance Council (BIRAC), New Delhi, India; Indian Institute of Technology Guwahati, INDIA

## Abstract

Coarse-grained simulations have emerged as a valuable tool in the study of large and complex biomolecular systems. These simulations, which use simplified models to represent complex biomolecules, reduce the computational cost of simulations and enable the study of larger systems for longer periods of time than traditional atomistic simulations. GROMACS is a widely used software package for performing coarse-grained simulations of biomolecules, and several force fields have been developed specifically for this purpose. In this protocol paper, we explore the advantages of using coarse-grained simulations in the study of biomolecular systems, focusing specifically on simulations performed using GROMACS. We discuss the force fields required for these simulations and the types of research questions that can be addressed using coarse-grained simulations. We also highlight the potential benefits of coarse-grained simulations for the development of new force fields and simulation methodologies. We then discuss the expected results from coarse-grained simulations using GROMACS and the various techniques that can be used to analyze these results. We explore the use of trajectory analysis tools, as well as thermodynamic and structural analysis techniques, to gain insight into the behavior of biomolecular systems.

## 1. Introduction

Molecular dynamics simulations have become a valuable tool in biological research for understanding the dynamics and behavior of biomolecules such as proteins, nucleic acids, and lipids [1]. However, simulating these large biomolecules can be computationally expensive due to the large number of atoms involved. This is where coarse-grained simulations come in. Coarse-grained simulations reduce the number of atoms in the system, which makes it possible to study larger and more complex biomolecular systems for longer periods of time than with atomistic simulations [2, 3]. In the following section, we will discuss coarse-grained simulation, the force fields required for such simulations, GROMACS [4] as a software package for performing these simulations, and the advantages of using coarse-grained simulations in biological research.

### 1.1 Coarse-grained simulation

In a coarse-grained simulation, groups of atoms are treated as a single entity called a "coarse-grained bead." By representing a group of atoms with a single bead, the number of atoms in the system is significantly reduced, which in turn reduces the computational cost of the simulation. The number of atoms that are grouped together to form a bead is determined by the level of coarse graining that is used [5]. A high level of coarse graining groups many atoms together to form a single bead, while a low level of coarse-graining groups fewer atoms together. The choice of the level of coarse graining depends on the size and complexity of the biomolecule being simulated and the research question being addressed [6].

### 1.2 Force fields for coarse-grained simulations

A force field is a set of mathematical equations that describes the interactions between atoms in a molecular system. In a coarse-grained simulation, force fields are used to describe the interactions between the coarse-grained beads. Force fields for coarse-grained simulations must balance accuracy with computational efficiency. Coarse-grained force fields typically have fewer parameters than atomistic force fields, which can make them faster to compute. However, coarse-grained force fields must also capture the essential features of the system being studied [7].

Several force fields have been developed specifically for coarse-grained simulations, including MARTINI [8], SIRAH [9], ELBA [10], and CGenFF [11]. MARTINI is a widely used coarse-grained force field that uses four to five heavy atoms to represent each amino acid or nucleotide, and groups together atoms that are chemically similar. SIRAH is a coarse-grained force field that uses a hybrid resolution approach, where the protein backbone and side chains are represented with different levels of coarse graining. ELBA is a coarse-grained force field that uses a combination of electrostatic and van der Waals interactions to represent the interactions between atoms. CGenFF is a general force field for simulating organic molecules, including proteins and nucleic acids. It uses a systematic coarse-graining approach to reduce the number of atoms in the simulation while still maintaining the accuracy of the simulation results.

### 1.3 GROMACS

GROMACS (GROningen MAchine for Chemical Simulations) is a software package that is widely used for molecular dynamics simulations of biomolecules. It is particularly useful for coarse-grained simulations because it is optimized for high-performance computing and can efficiently simulate large biomolecular systems. GROMACS has a variety of built-in force fields, including several specifically designed for coarse-grained simulations, such as MARTINI and SIRAH. GROMACS also has a user-friendly interface that makes it easy to set up and run simulations.

## 2. Methods

### 2.1 Protocol for the development of coarse-grained structures for macromolecular simulation using GROMACS

The protocol described CG simulation of biomolecules in this article is published on protocols.io [12]. https://dx.doi.org/10.17504/protocols.io.kxygx92rdg8j/v1 A detailed video tutorial and the required files is available at https://youtu.be/QMR4f4eRSbs. The video tutorial is available in 1080p HD resolution and we kindly request so set the resolution to best quality.

The detailed protocol is provided as S1 File.

### 2.2 Protocol for coarse grained simulation of protein ligand system using GROMACS

The protocol described CG simulation of protein ligand system in this article is published on protocols.io [13]. https://dx.doi.org/10.17504/protocols.io.3byl4jm8rlo5/v1

A detailed video tutorial and the required files is available at https://youtu.be/xjfbA1G3PIM. The video tutorial is available in 1080p HD and 2160p HD resolution and we kindly request so set the resolution to best quality.

The detailed protocol is provided as S2 File.

## 3. Results

The expected results from coarse-grained simulations using GROMACS will depend on the specific system being studied and the research question being asked. However, in general, coarse-grained simulations can provide insight into the collective behavior of biomolecules, the structural and thermodynamic properties of biomolecules, and the interactions between biomolecules and their environment.

### 1. Conversion of All atom model to coarse grained model

**Fig 1:**
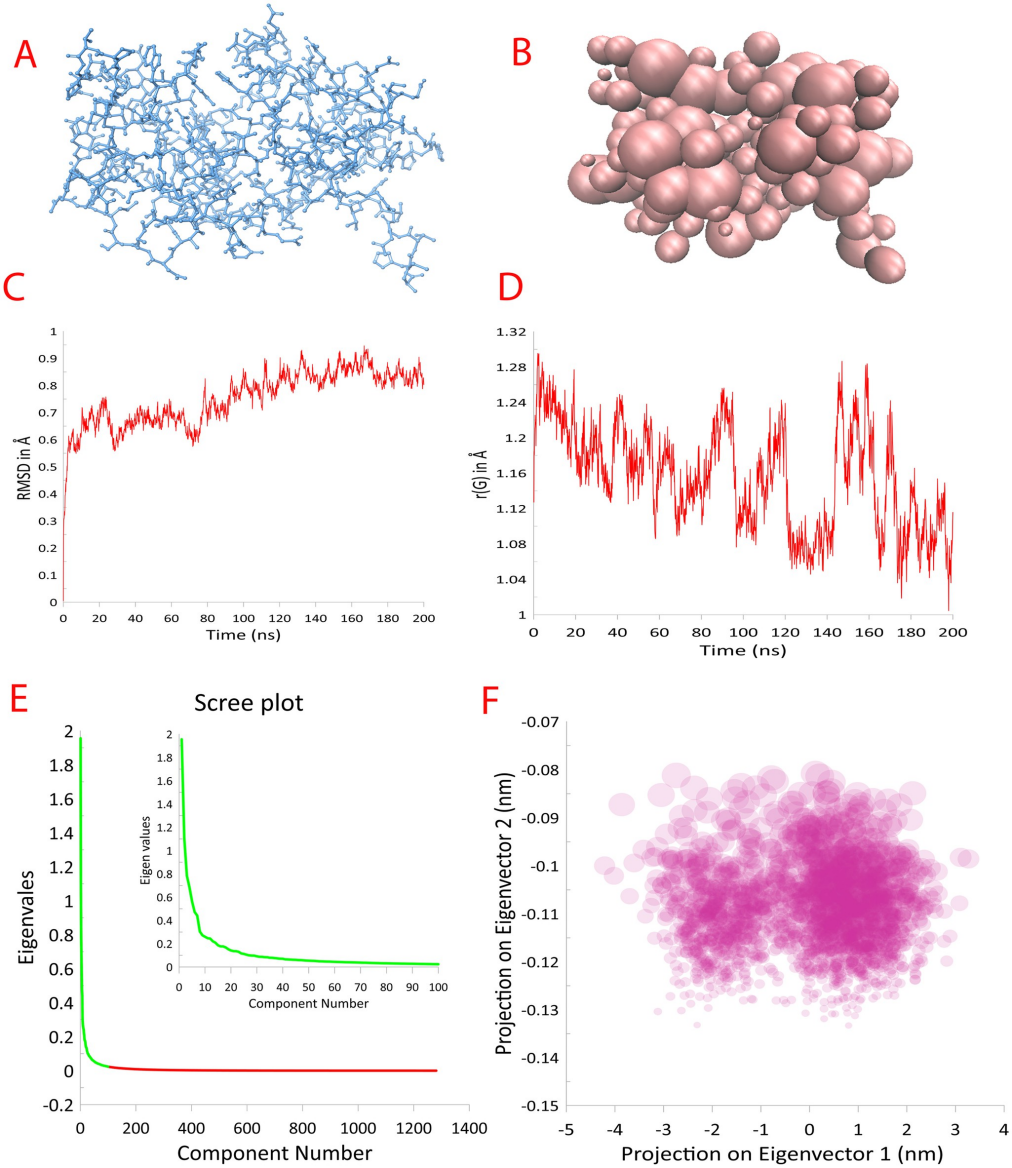
Conversion of AA model and expected results from CG simulation and analysis. A) A protein all atom model 3D structure in wireframe representation colored in blue. B) The protein all atom model converted to CG model with bead representation. C) The RMSD plot after 200ns CG simulation. D) The plot of radius of gyration versus time during protein ligand CG simulation. E) The scree plot with eigenvalues vs component number. The green section highlighted in the main plot is enhanced in the secondary in-plot to show the elbow region of the plot. F) PCA plot of projection of eigenvectors 1 versus eigenvectors 2 in nm.

The conversion of an all-atom model to a coarse-grained model can provide a basis for the development of simplified models that can be used in drug discovery and other applications. By reducing the number of particles in the system and simplifying the force field parameters, coarse-grained models can be used to rapidly screen large numbers of potential drug candidates and to design new molecules with improved binding properties. Please refer to Fig 1A and 1B.

Fig 1A represents the all-atom model of protein AAC’2 (PDB ID: 1M4I) [14] was retrieved from RCSB-PDB [15]. The structure is shown in wireframe representation colored in blue.

In Fig 1B, the structure was converted to coarse grained model and shown in beads representation.

### 2. Performing coarse grained simulation on apo protein or protein ligand complex

In the case of an apo protein, coarse-grained simulations can provide information on the dynamics and stability of the protein structure, as well as the conformational changes that occur during protein folding or unfolding. Coarse-grained simulations can also be used to study the interactions between different regions of the protein, such as the formation of secondary structures and the behavior of disordered regions.

In the case of a protein-ligand complex, coarse-grained simulations can provide insight into the binding mechanism and the thermodynamics of ligand binding. These simulations can be used to calculate the binding free energy and the potential of mean force (PMF) of the protein-ligand interaction.

Radius of gyration (Rg) is a measure of the overall size and shape of a molecule. It is calculated as the root mean square (RMS) distance of all atoms in the molecule from the center of mass. A small Rg indicates a compact molecule, while a large Rg indicates an extended molecule.

The Rg can be used to study the folding and unfolding of proteins, the binding of ligands to proteins, and the dynamics of biomolecular systems. For example, a decrease in the Rg of a protein during MD simulation can indicate that the protein is folding. An increase in the Rg of a protein can indicate that the protein is unfolding.

### 3. Analysis of results from coarse grained simulation

In terms of analyzing the results of coarse-grained simulations, there are several techniques that can be used. One commonly used technique is to analyze the trajectories generated by the simulation using various tools available in GROMACS. For example, the root-mean-square deviation (RMSD) and root-mean-square fluctuation (RMSF) can be calculated to measure the structural stability of the system over time. The RMSD was found to be less than 1 Å. The RMSD plot of C-alpha versus time is provided in Fig 1C.

Rg of the protein structure over a period of simulation period was calculated. The Rg value of the protein was found to be decreasing over the simulation time period from 1.28 Å to 1.12 Å signifying the increase in compactness and folding of the protein. The plot of gyration values versus time is provided in Fig 1D.

The total expansion of a protein during different simulations is reflected by the principal component analysis (PCA) or essential dynamics (ED) [16]. Using the gmx covar module, the dynamics of protein with regard to the backbone were computed in this approach. A protein’s large-scale average motion is found via PCA, which also reveals the structures that underlie the atomic fluctuations [17]. The system’s overall motility is gauged by the sum of the eigenvalues [18]. During the PCA calculations, the protein’s root mean square atomic variations were also noted. Further resolution of the Eigenvector components into the x, y, and z directions.

The Fig 1E, represents the scree plot of eigenvalues. The green section of the plot is highlighted in the enhanced section to show the elbow region. Fig 1F, highlights the PCA plot of projection of eigenvectors 1 versus eigenvectors 2 in nm.

In addition, coarse-grained simulations can be used to generate structural models of biomolecular complexes, which can be analyzed using various tools such as molecular docking and molecular dynamics simulations.

Overall, the analysis of results from coarse-grained simulations using GROMACS can provide valuable insight into the behavior of biomolecular systems and can inform the design of new experiments and therapies targeting specific biomolecules or molecular interactions.

## 4. Test cases

### 1. Identification of active site/ligand binding site

Coarse-grained simulation can be used to identify the active site of an enzyme. The active site is the region of the enzyme where the substrate binds and is converted into the product. In a coarse-grained simulation, the active site can be identified by looking for regions of the molecule that are highly mobile and that interact with the substrate.

Coarse-grained simulation is a powerful tool for studying the behavior of proteins. It can be used to identify the active site of an enzyme, to study the mechanism of enzyme catalysis, and to understand how proteins interact with other molecules.

In the current study, we have used AAC2 from is an aminoglycoside 2’-N-acetyltransferase, which means that it can add an acetyl group to the 2’-hydroxyl group of aminoglycoside antibiotics [19]. This modification makes the antibiotics inactive, which can lead to resistance to treatment [20]. The aac(2’)-Ic gene is found in all strains of *M*. *tuberculosis*, and it is one of the most common genes associated with drug resistance.

Since AAC2 has known interactions with aminoglycosides we have kanamycin as a test case to identify as potential active site.

Using the above-mentioned protocol, we run CG simulations for 200 nanoseconds (ns), to identify and define the active site by calculating the occupancy of kanamycin.

**Fig 2:**
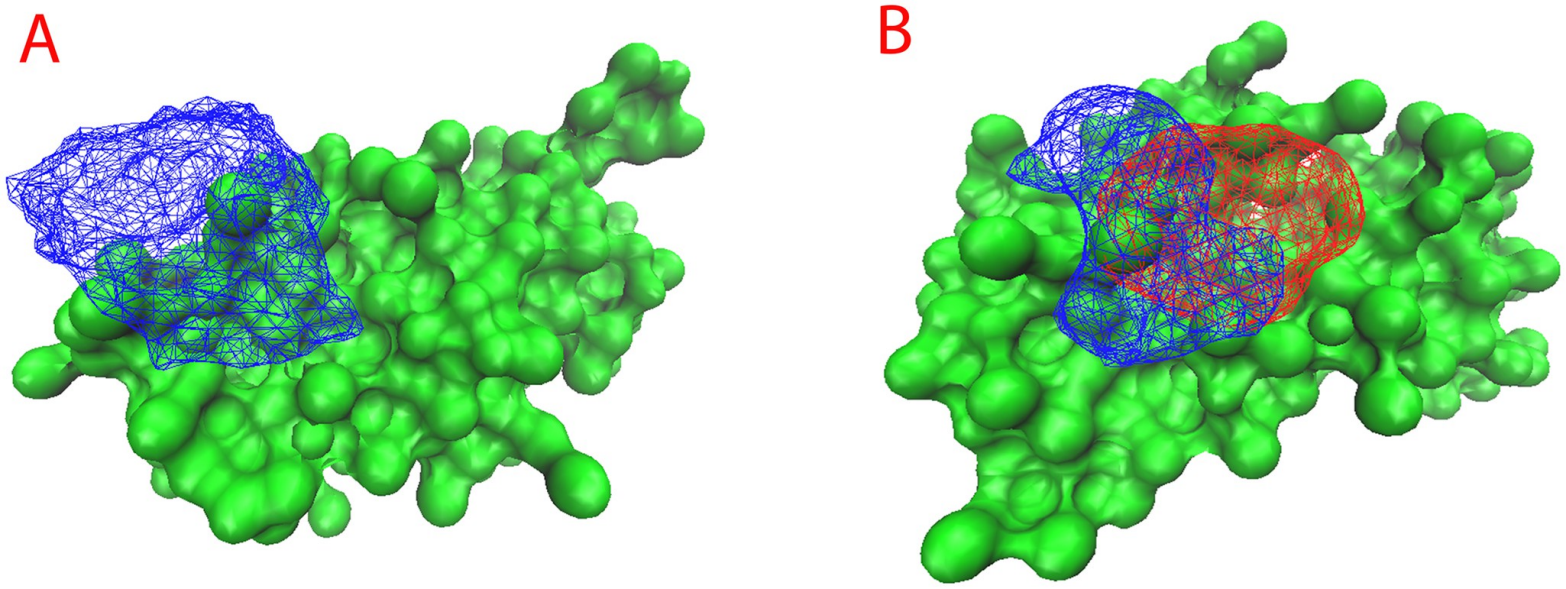
Occupancy map of compounds in protein compound system. A) The occupancy map of kanamycin on the surface of protein (green) in shown in blue. B) The occupancy map of kanamycin on the surface of protein (green) in shown in blue and streptomycin in red.

The plot can be seen in Fig 2A with the protein shown in green colored surface representation and the occupancy of kanamycin shown in blue mesh representation.

### 2. Insights on the “ligand preference” during competitive binding with long timescale simulation

In the case of competitive binding, CG simulations can be used to verify the ability of two or more compounds in the defined active site. This is an enhanced method to gain insights into the binding ability of compounds compared to the combination of molecular docking and molecular dynamic simulations.

In the current study, we have used streptomycin and kanamycin as potential aminoglycosides and verify the binding ability with AAC2. Using the above-mentioned protocol, we run CG simulations for 2 microseconds (μs), we identify the binding and retention ability of the compounds within the binding pocket. Occupancy map is shown in Fig 2B, the protein shown in green colored surface representation and the occupancy of kanamycin shown in blue mesh representation and streptomycin in red mesh representation. The results show the competitive ability of the compounds to interact with the binding pocket and over lapping with each other, which corresponds to the ability of the protein to bind to both the aminoglycosides.

## 5. Conclusion

Coarse-grained simulations are a valuable tool in biological research for studying large and complex biomolecular systems. Coarse-grained simulations reduce the computational cost of simulations, which makes it possible to study larger and more complex systems for longer periods of time than with atomistic simulations. Coarse-grained simulations can provide insight into the collective behavior of biomolecules, the structural and thermodynamic properties of biomolecules, and the interactions between biomolecules and their environment. GROMACS is a widely used software package for performing coarse-grained simulations of biomolecules, and several force fields have been developed specifically for coarse-grained simulations. Coarse-grained simulations are a rapidly growing field of research, and they have the potential to revolutionize our understanding of biological systems.

## Supporting information

S1 File A detailed step by step protocol on the development of coarse-grained structures for macromolecular simulation using GROMACS. (PDF)

S2 File A detailed step by step protocol on coarse grained simulation of protein ligand system using GROMACS. (PDF)
