# Supplementary material for: Protocol for the development of coarse-grained structures for macromolecular simulation using GROMACS
Source: PLoS One. 2023 Aug 3;18(8):e0288264. doi: 10.1371/journal.pone.0288264 (PMC10399882; doi:10.1371/journal.pone.0288264)
Supplement: S1 File — (PDF) [file pone.0288264.s001.pdf]

protocols.io

Protocol for the development of coarse-grained structures for macromolecular simulation using GROMACS

M Purushotham Rao[1], Akshay Uttarkar[1], Vidya Niranjan[1]

[1]R V College of Engineering

Vidya Niranjan

MAR 21, 2023

OPEN ACCESS

DOI: dx.doi.org/10.17504/protocols.io.kxygx92rdg8j/v1

Protocol Citation: M Purushotham Rao, Akshay Uttarkar, Vidya Niranjan 2023. Protocol for the development of coarse-grained structures for macromolecular simulation using GROMACS. **protocols.io** https://dx.doi.org/10.17504/protocols.io.kxygx92rdg8j/v1

License: This is an open access protocol distributed under the terms of the Creative Commons Attribution License, which permits unrestricted use, distribution, and reproduction in any medium, provided the original author and source are credited

Protocol status: Working
We use this protocol and it's working

Created: Feb 28, 2023

Last Modified: Mar 21, 2023

PROTOCOL integer ID: 77756

Keywords: Martini, Coarse grain, Molecular Simulation

ABSTRACT

This paper presents a protocol for the development of coarse-grained (CG) structures for macromolecular simulation using the GROMACS software. CG models are widely used in molecular simulations due to their computational efficiency, which allows for the study of large and complex systems. The protocol described here outlines the steps necessary for the creation of CG structures, including the selection of appropriate beads, mapping of the CG beads onto the atomistic structure, and the parameterization of the CG model. The protocol also includes guidelines for validating the accuracy of the CG model, as well as recommendations for future improvements in CG model development. The described protocol will be useful for researchers interested in the development of CG models for macromolecular simulations using GROMACS.

GUIDELINES

Commands are indicated in bold letters

MATERIALS

https://drive.google.com/file/d/1YDJV2hKtZ5dJrl8S6A_4AFdTt_lVJFMv/view?usp=sharing
https://github.com/MPurushothamRao/miscellaneous
https://drive.google.com/file/d/1if8nCmmOAXT-ZTEQGu2ctG3bcgaQayPi/view?usp=sharing

SAFETY WARNINGS

Ensure all the requirements are satisfied for tools like Gromacs, Dssp.
If there are more warning while running gromas check for their impact, if its not harmful. Ignore it using maxwarn

BEFORE START INSTRUCTIONS

A basic understanding on gromacs and simulations.
For visual assistance refer to https://youtu.be/QMR4f4eBSbs

DOWNLOAD NECESSARY PROTEIN

1 DOWNLOAD THE PDB FILE FROM https://www.rcsb.org/
Here, in this tutorial DUSP28 https://www.rcsb.org/structure/5Y15 is used.
Preprocess the pdb to remove all ions and B chain or can obtained from here https://drive.google.com/file/d/1YDJV2hKtZ5dJrl8S6A_4AFdTt_IVJFMv/view?usp=sharing

DOWNLOAD NECESSARY SOFTWARE AND FILES

2 Martinize python script http://cgmartini.nl/index.php/tools2/proteins-and-bilayers/204-martinize
Martini itp file required version http://cgmartini.nl/index.php/force-field-parameters/particle-definitions
Martinin ions itp file http://cgmartini.nl/index.php/force-field-parameters/ions
Dssp Executable https://github.com/cmbi/dssp - use source 2.3 version
dssp to ssd python script (Optional) https://github.com/MPurushothamRao/miscellaneous
Gromacs type this in terminal- sudo apt-get install gromacs
mdp files https://drive.google.com/file/d/1if8nCmmOAXT-ZTEQGu2ctG3bcgaQayPi/view?usp=sharing
Non-polarised water or Polarised water gro files http://cgmartini.nl/index.php/downloads/example-applications/63-pure-water-solvent
VMD https://www.ks.uiuc.edu/Development/Download/download.cgi?PackageName=VMD
XMGRACE type this in terminal- sudo apt-get install grace
Commands shell script https://github.com/MPurushothamRao/miscellaneous

COARSE GRAINING OF PROTEIN

3 change dssp executable path and required force field and use python3 martinize.py -h for help
python3 martinize.py -f 5y15_processed.pdb -o single-5y15.top -x 5y15-CG.pdb -dssp /usr/local/bin/mkdssp -p backbone -ff martini22
or use ssd file as input
mkdssp -i 5y15.pdb -o 5y15.dssp
to conver dssp file to ssd file
python3 dssp2ssd.py -i 5y15.dssp -o 5y15.ssd
python3 martinize.py -f 5y15_processed.pdb -o single-5y15.top -x 5y15-CG.pdb -ss 5y15.ssd -p backbone -ff martini22
Here we have used second method.

```
(base) rvce-bt-06@rvcebt06-HP-280-G3-MT:~/Desktop/purushotham_bbt21/single_cheq$ mkdssp -i 1UBQ.pdb -o 1UBQ.dssp
(base) rvce-bt-06@rvcebt06-HP-280-G3-MT:~/Desktop/purushotham_bbt21/single_cheq$ python3 dssp2ssd.py -i 1UBQ.dssp -o 1UBQ.ssd
(base) rvce-bt-06@rvcebt06-HP-280-G3-MT:~/Desktop/purushotham_bbt21/single_cheq$ python martinize.py -f 1UBQ.pdb -o single-ubq.top -x 1UBQ-CG.
pdb -ss 1UBQ.ssd -p backbone -ff martini22
INFO       MARTINIZE, script version 2.6_3
INFO       If you use this script please cite:
INFO       de Jong et al., J. Chem. Theory Comput., 2013, DOI:10.1021/ct300646g
INFO       Chain termini will be charged
INFO       Residues at chain brakes will not be charged
INFO       The martini22 forcefield will be used.
INFO       Local elastic bonds will be used for extended regions.
INFO       Position restraints will be generated.
WARNING    Position restraints are only enabled if -DPOSRES is set in the MDP file
INFO       Read input structure from file.
INFO       Input structure is a PDB file.
INFO       Found 2 chains:
INFO          1:   A (), 602 atoms in 76 residues.
INFO          2:   A (), 58 atoms in 58 residues.
INFO       Removing 58 water molecules (chain A).
INFO       Total size of the system: 76 residues.
INFO       Will read secondary structure from file (assuming Gromacs ssdump).
INFO       Writing coarse grained structure.
INFO       (Average) Secondary structure has been determined (see head of .itp-file).
INFO       Created coarsegrained topology
INFO       Written 1 ITP file
INFO       Output contains 1 molecules:
INFO          1->  Protein_A (chain  A)
INFO       Written topology files
INFO       Note: Cysteine bonds are 0.24 nm constraints, instead of the published 0.39nm/5000kJ/mol.

        There you are. One MARTINI. Shaken, not stirred.


 Why don't you get out of that wet coat and into a dry martini?
                                                    --Robert Benchley
```

Output of Martinizing (Coarse graining) of the protein

4 to change name of martini itp file in toplogy, for what you have selected in above step

sed -i -e 's/martini.itp/martini_v2.2.itp/' single-ubq.top

```
#include "martini_v2.2.itp"


#include "Protein_A.itp"

[ system ]
; name
Martini system from 1UBQ.pdb

[ molecules ]
; name        number
Protein_A        1
```

Snap of topology file after above command

SYSTEM SETUP

5 Setup Periodic box

gmx editconf -f 1UBQ-CG.pdb -o 1UBQ-CG.gro -d 1.0 -c -bt dodecahedron

```
Command line:
  gmx editconf -f 1UBQ-CG.pdb -o 1UBQ-CG.gro -d 1.0 -c -bt dodecahedron

Note that major changes are planned in future for editconf, to improve usability and utility.
Read 163 atoms
Volume: 62.9497 nm^3, corresponds to roughly 28300 electrons
No velocities found
    system size :  2.763  2.966  3.382 (nm)
    diameter    :  4.224               (nm)
    center      :  2.999  2.891  1.522 (nm)
    box vectors :  5.084  4.277  2.895 (nm)
    box angles  :  90.00  90.00  90.00 (degrees)
    box volume  :  62.95               (nm^3)
    shift       :  1.670  1.778  0.679 (nm)
new center      :  4.668  4.668  2.201 (nm)
new box vectors :  6.224  6.224  6.224 (nm)
new box angles  :  60.00  60.00  90.00 (degrees)
new box volume  :  170.53              (nm^3)

GROMACS reminds you: "I don't want to achieve immortality through my work... I want to achieve it through not dying!" (Woody Allen)
```

Output after addition of Box

6 To minimise the coarse_grained structure in vaccum

gmx grompp -f em_vac.mdp -c 1UBQ-CG.gro -p single-ubq.top -o em_vac.tpr

6.1 **gmx mdrun -deffnm em_vac -v**

```
Step=  100, Dmax= 7.2e-03 nm, Epot= -3.21980e+03 Fmax= 4.83235e+02, atom= 56
Energy minimization reached the maximum number of steps before the forces
reached the requested precision Fmax < 10.

writing lowest energy coordinates.

Steepest Descents did not converge to Fmax < 10 in 101 steps.
Potential Energy  = -3.2234973e+03
Maximum force     =  1.3524817e+02 on atom 63
Norm of force     =  3.5189513e+01

GROMACS reminds you: "Stay Cool, This is a Robbery" (Pulp Fiction)
```

After energy minimization in Vacuum

7 Solvate the protein

gmx solvate -cp em_vac.gro -cs water.gro -radius 0.21 -o solvated.gro

7.1 To add number of water molecules into toplogy file for polarised water divide count by 3

cp single-ubq.top system.top

count=$(grep -c “W” solvated.gro | tr -d ‘\n’)

echo -e “\nW $count” >> system.top

```
Generating solvent configuration
Will generate new solvent configuration of 2x2x2 boxes
Solvent box contains 1926 atoms in 1926 residues
Removed 643 solvent atoms due to solvent-solvent overlap
Removed 117 solvent atoms due to solute-solvent overlap
Sorting configuration
Found 1 molecule type:
      W (   1 atoms):  1166 residues
Generated solvent containing 1166 atoms in 1166 residues
Writing generated configuration to solvated.gro

Output configuration contains 1329 atoms in 1242 residues
Volume                 :     170.528 (nm^3)
Density                :      2122.5 (g/l)
Number of solvent molecules:   1166


GROMACS reminds you: "Hangout In the Suburbs If You've Got the Guts" (Urban Dance Squad)

(base) rvce-bt-06@rvcebt06-HP-280-G3-MT:~/Desktop/purushotham_bbt21/single_cheq$ cp single-ubq.top system.top
(base) rvce-bt-06@rvcebt06-HP-280-G3-MT:~/Desktop/purushotham_bbt21/single_cheq$ count=$(grep -c "W" solvated.gro | tr -d '\n')
(base) rvce-bt-06@rvcebt06-HP-280-G3-MT:~/Desktop/purushotham_bbt21/single_cheq$ echo -e "\nW    $count" >> system.top
```

Addition of water molecules and making system topology files

8 Add ions (optional to neutralise or addition ions)

gmx grompp -f ions.mdp -c solvated.gro -p system.top -o ions.tpr

gmx genion -s ions.tpr -o ions.gro -p protein.top -pname NA+ -nname CL- -conc 0.1 -neutral

we have not added here but in the video its shown how to add.

SIMULATION

9 Energy minimisation

gmx grompp -f em.mdp -c solvated.gro -r solvated.gro -p system.top -o em.tpr -maxwarn 1

maxwarn is because there is an mismatch of atom names but all the atoms are

present

9.1 **gmx mdrun -deffnm em -v**

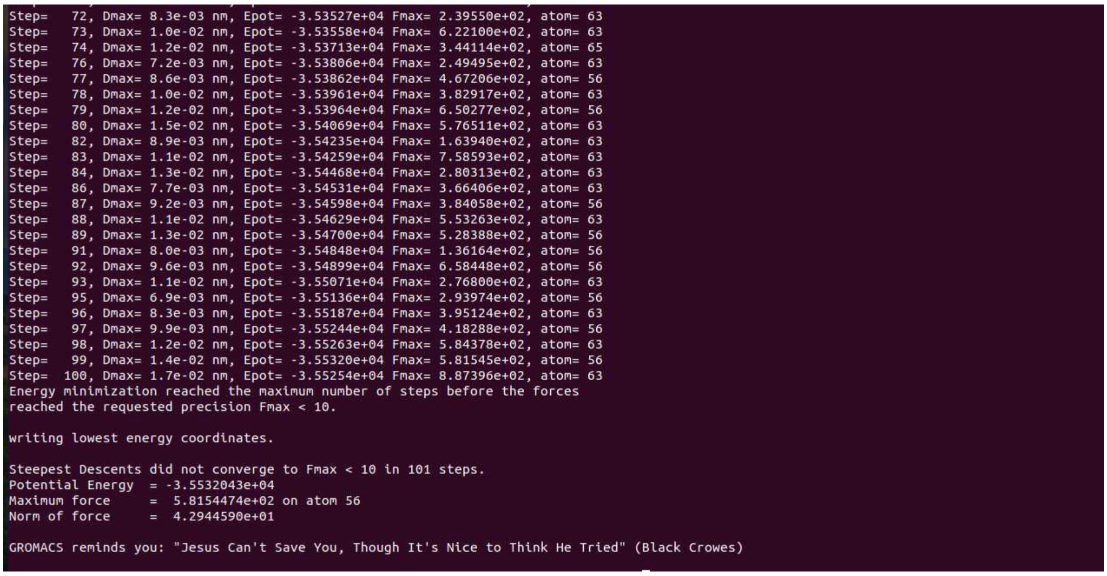

```
Step=   72, Dmax= 8.3e-03 nm, Epot= -3.53527e+04 Fmax= 2.39550e+02, atom= 63
Step=   73, Dmax= 1.0e-02 nm, Epot= -3.53558e+04 Fmax= 6.22100e+02, atom= 63
Step=   74, Dmax= 1.2e-02 nm, Epot= -3.53713e+04 Fmax= 3.44114e+02, atom= 65
Step=   76, Dmax= 7.2e-03 nm, Epot= -3.53806e+04 Fmax= 2.49495e+02, atom= 63
Step=   77, Dmax= 8.6e-03 nm, Epot= -3.53862e+04 Fmax= 4.67206e+02, atom= 56
Step=   78, Dmax= 1.0e-02 nm, Epot= -3.53961e+04 Fmax= 3.82917e+02, atom= 63
Step=   79, Dmax= 1.2e-02 nm, Epot= -3.53964e+04 Fmax= 6.50277e+02, atom= 56
Step=   80, Dmax= 1.5e-02 nm, Epot= -3.54069e+04 Fmax= 5.76511e+02, atom= 63
Step=   82, Dmax= 8.9e-03 nm, Epot= -3.54235e+04 Fmax= 1.63940e+02, atom= 63
Step=   83, Dmax= 1.1e-02 nm, Epot= -3.54259e+04 Fmax= 7.58593e+02, atom= 63
Step=   84, Dmax= 1.3e-02 nm, Epot= -3.54468e+04 Fmax= 2.80313e+02, atom= 63
Step=   86, Dmax= 7.7e-03 nm, Epot= -3.54531e+04 Fmax= 3.66406e+02, atom= 63
Step=   87, Dmax= 9.2e-03 nm, Epot= -3.54598e+04 Fmax= 3.84058e+02, atom= 56
Step=   88, Dmax= 1.1e-02 nm, Epot= -3.54629e+04 Fmax= 5.53263e+02, atom= 63
Step=   89, Dmax= 1.3e-02 nm, Epot= -3.54700e+04 Fmax= 5.28388e+02, atom= 56
Step=   91, Dmax= 8.0e-03 nm, Epot= -3.54848e+04 Fmax= 1.36164e+02, atom= 56
Step=   92, Dmax= 9.6e-03 nm, Epot= -3.54899e+04 Fmax= 6.58448e+02, atom= 56
Step=   93, Dmax= 1.1e-02 nm, Epot= -3.55071e+04 Fmax= 2.76800e+02, atom= 63
Step=   95, Dmax= 6.9e-03 nm, Epot= -3.55136e+04 Fmax= 2.93974e+02, atom= 56
Step=   96, Dmax= 8.3e-03 nm, Epot= -3.55187e+04 Fmax= 3.95124e+02, atom= 63
Step=   97, Dmax= 9.9e-03 nm, Epot= -3.55244e+04 Fmax= 4.18288e+02, atom= 56
Step=   98, Dmax= 1.2e-02 nm, Epot= -3.55263e+04 Fmax= 5.84378e+02, atom= 63
Step=   99, Dmax= 1.4e-02 nm, Epot= -3.55320e+04 Fmax= 5.81545e+02, atom= 56
Step=  100, Dmax= 1.7e-02 nm, Epot= -3.55254e+04 Fmax= 8.87396e+02, atom= 63
Energy minimization reached the maximum number of steps before the forces
reached the requested precision Fmax < 10.

writing lowest energy coordinates.

Steepest Descents did not converge to Fmax < 10 in 101 steps.
Potential Energy  = -3.5532043e+04
Maximum force     =  5.8154474e+02 on atom 56
Norm of force     =  4.2944590e+01

GROMACS reminds you: "Jesus Can't Save You, Though It's Nice to Think He Tried" (Black Crowes)
```

Energy Minimisation

10 NVT equilibration

gmx grompp -f nvt.mdp -c em.gro -r em.gro -p system.top -o nvt.tpr

10.1 **gmx mdrun -deffnm nvt -v**

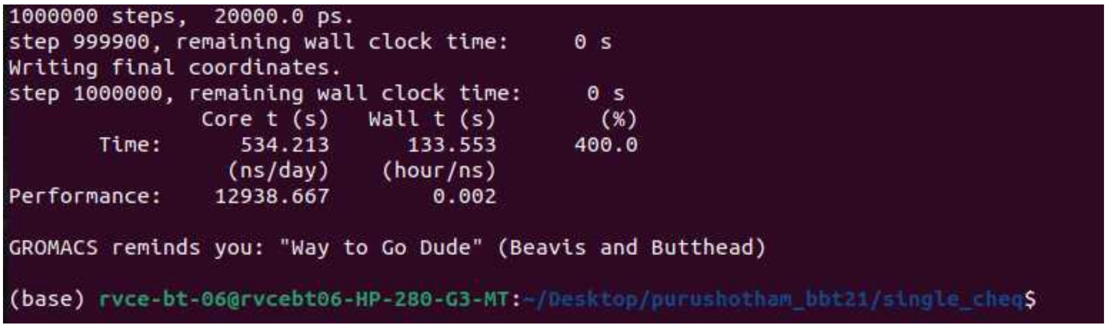

```
1000000 steps,  20000.0 ps.
step 999900, remaining wall clock time:     0 s
Writing final coordinates.
step 1000000, remaining wall clock time:     0 s
               Core t (s)   Wall t (s)        (%)
       Time:      534.213      133.553      400.0
                 (ns/day)    (hour/ns)
Performance:    12938.667        0.002

GROMACS reminds you: "Way to Go Dude" (Beavis and Butthead)

(base) rvce-bt-06@rvcebt06-HP-280-G3-MT:~/Desktop/purushotham_bbt21/single_cheq$
```

NVT equilibration for 20ns

11 NPT equilibration

gmx grompp -f npt.mdp -c nvt.gro -r nvt.gro -p system.top -o npt.tpr

11.1 **gmx mdrun -deffnm npt -v**

```
1000000 steps,  20000.0 ps.
step 999900, remaining wall clock time:     0 s
Writing final coordinates.
step 1000000, remaining wall clock time:     0 s
               Core t (s)   Wall t (s)        (%)
       Time:      544.539      136.135      400.0
                 (ns/day)    (hour/ns)
Performance:    12693.311        0.002

GROMACS reminds you: "Do the Dog On the Ground" (Red Hot Chili Peppers)
```

NPT equilibration for 20ns

12 MD run

gmx grompp -f md.mdp -c npt.gro -p system.top -o md.tpr

12.1 **gmx mdrun -deffnm md -v**

`

```
Compiled SIMD: SSE4.1, but for this host/run AVX2_256 might be better (see
log).
Reading file md.tpr, VERSION 2021.4-Ubuntu-2021.4-2 (single precision)
Changing nstlist from 20 to 25, rlist from 1.218 to 1.267

Using 1 MPI thread
Using 4 OpenMP threads

starting mdrun 'Martini system from 1UBQ.pdb'
10000000 steps, 200000.0 ps.
step 9999900, remaining wall clock time:     0 s
Writing final coordinates.
step 10000000, remaining wall clock time:     0 s
               Core t (s)   Wall t (s)        (%)
       Time:     5309.617     1327.404      400.0
                 (ns/day)    (hour/ns)
Performance:    13017.890        0.002

GROMACS reminds you: "It Was My Pleasure" (Pulp Fiction)
```

Production run for 200 ns

ANALYSIS

13 Analysis

Before analysis conect command should be used to show bonds in visualisation software and also pbc should be removed

echo 1 1 | gmx trjconv -f md.gro -s md.tpr -o recentered_traj.gro -pbc mol -center
echo 1 | gmx trjconv -f recentered_traj.gro -s md.tpr -conect -o connected_traj.pdb
echo 1 1 | gmx trjconv -f md.xtc -s md.tpr -o recentered_traj.xtc -pbc mol -center
sed -i '/ENDMDL/d' connected_traj.pdb
to visualize
vmd recentered_traj.xtc connected_traj.pdb

Video shows protein over 20ns

13.1 Calculation RMSD and radius of Gyration and plotting using XMGRACE

echo 1 1 | gmx rms -s md.tpr -f recentered_traj.xtc -o rmsd.xvg

xmgrace rmsd.xvg

echo 1 | gmx gyrate -s md.tpr -f recentered_traj.xtc -o gyrate.xvg

xmgrace gyrate.xvg

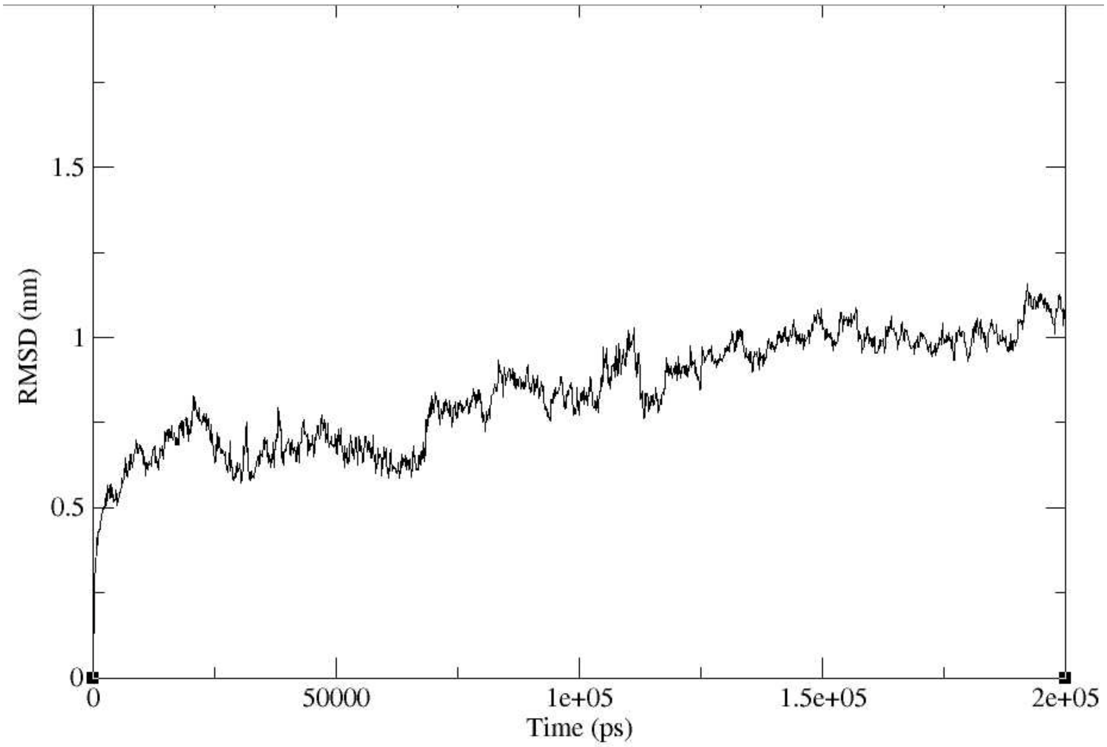

RMSD plot Distance nm vs Time ps

14 Step 2- 12 can be automated using a shell script

sh commands.sh

15 Video of tutorial

https://youtu.be/QMR4f4eRSbs
