# Supplementary material for: Protocol for the development of coarse-grained structures for macromolecular simulation using GROMACS
Source: PLoS One. 2023 Aug 3;18(8):e0288264. doi: 10.1371/journal.pone.0288264 (PMC10399882; doi:10.1371/journal.pone.0288264)
Supplement: S2 File — (PDF) [file pone.0288264.s002.pdf]

Protocol for coarse grained simulation of protein ligand system using GROMACS

M Purushotham Rao[1], Akshay Uttarkar[1], Vidya Niranjan[1]

[1]R V College of Engineering

Vidya Niranjan

Protocol Info: M Purushotham Rao, Akshay Uttarkar, Vidya Niranjan . Protocol for coarse grained simulation of protein ligand system using GROMACS. **protocols.io** https://protocols.io/view/protocol-for-coarse-grained-simulation-of-protein-cu4dwys6

Created: Jun 02, 2023

Last Modified: Jun 03, 2023

PROTOCOL integer ID: 82789

ABSTRACT

Coarse-grained (CG) simulations are a powerful tool for studying the behavior of biomolecular systems. They are becoming increasingly important tools for drug discovery, as they can be used to study a wide variety of systems over long timescales.
CG simulations are faster than all-atom MD simulations, which allows researchers to study larger systems over longer timescales. They can also be used to study systems that are too large or too complex to be studied with all-atom MD simulations. Additionally, CG simulations can be used to study systems that are difficult or impossible to study experimentally. CG simulations are typically 100-1000 times faster than all-atom MD simulations.
This protocol provides steps along with a video tutorial perform CG simulation for protein ligand system. The advantages and test cases are but not limited to identification of active site, detection of cryptic pockets on protein, competitive binding between two or more ligands and many more
The link the video tutorial is available in https://youtu.be/xjfbA1G3PIM

BEFORE START INSTRUCTIONS

A basic understanding on gromacs and simulations.
For visual assistance refer to https://youtu.be/xjfbA1G3PIM
PART-1
The tutorial on Protocol for the development of coarse-grained structures for macro molecular simulation using GROMACS is available at https://protocols.io/view/protocol-for-the-development-of-coarse-grained-str-cp64vrgw.html and visual assistance for the same at https://youtu.be/QMR4f4eRSbs

1 Preprocessing of protein
Removal of Heteroatoms and if required removing other chains
grep "^ATOM" 1m4i.pdb > 1m4i_clean.pdb | grep " A " 1m4i_clean.pdb > 1m4i_singlechain.pdb

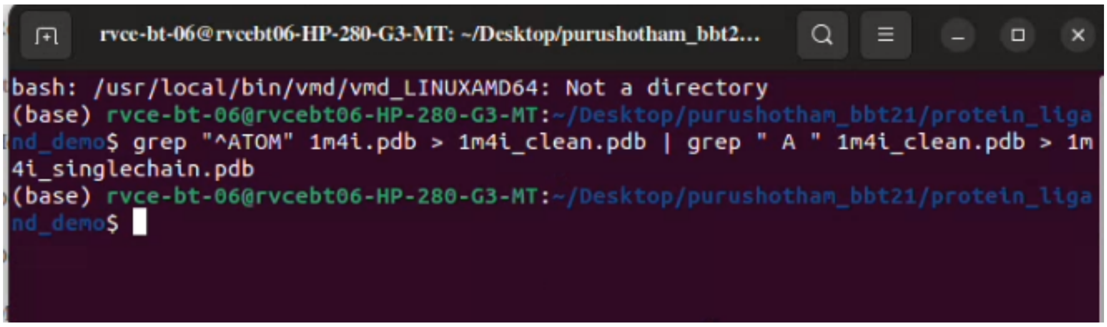

Preprocessing of protein

2 Finding secondary structure of 1m4i (AAC2)

mkdssp -i 1m4i.pdb -o 1m4i.dssp

python dssp2ssd.py -i 1m4i.dssp -o 1m4i.ssd

Second line of ssd file contains required secondary structure of 1m4i chain A

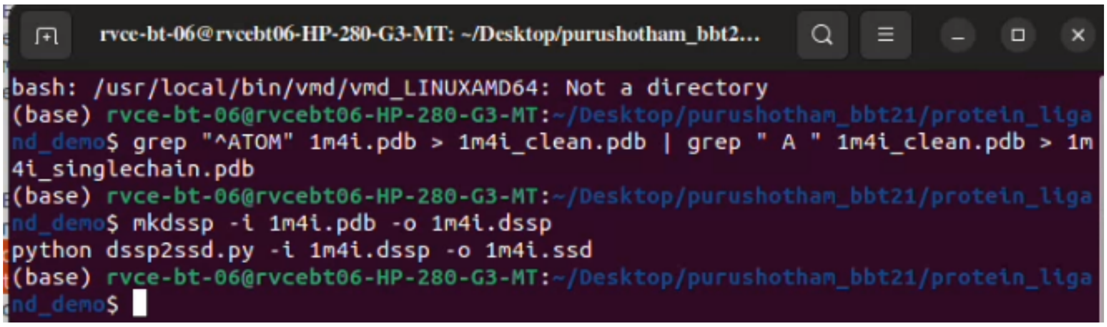

Secondary structure of 1m4i

3 Martinize: Convert All atomic model of AAC2 to Coarse grain model

martinize2 -f 1m4i_singlechain.pdb -o 1m4i_ONLY.top -x 1m4i_CG.pdb -ss
CCCCTTCCEEEEGGGCCHHHHHHHHHHHHHHTTTCCCHHHHHHTCSSEEEEEEETTEEE
EEEEEEEEEEEETTEEEEEEEEEEEEECGGGTTSSHHHHHHHHHHHHHHHCSEEEEECCTT
THHHHHHTTCEECCSCEEEEETTEEEECGGGTTTEEEEESSCCCCTTSCEEEECCSSCCC
-p backbone -ff martini3001 -elastic -ef 500.0 -el 0.5 -eu 0.8 -scfix -cy auto

```
rvce-bt-06@rvcebt06-HP-280-G3-MT: ~/Desktop/purushotham_bbt2...
    INFO - general - Identified the modifications ['N-ter'] on residues ['MET1',
 'MET1', 'MET1', 'MET1']
    INFO - general - Identified the modifications ['C-ter'] on residues ['TRP181
', 'TRP181', 'TRP181']
    INFO - step - Read input.
    INFO - step - Creating the graph at the target resolution.
    INFO - general - Applying modification mapping ('C-ter',)
    INFO - general - Applying modification mapping ('N-ter',)
    INFO - step - Averaging the coordinates.
    INFO - step - Applying the links.
    INFO - step - Placing the charge dummies.
    INFO - step - Applying position restraints.
    INFO - step - Setting the rubber bands.
    INFO - step - Writing output.
    INFO - general - Please cite: Souza, P C T; Alessandri, R; Barnoud, J; Thall
mair, S; Faustino, I; Grünewald, F; Patmanidis, I; Abdizadeh, H; Bruininks, B M
H; Wassenaar, T A; Kroon, P C; Melcr, J; Nieto, V; Corradi, V; Khan, H M; Domański, J; Javanainen, M; Martinez-Seara, H; Reuter, N; Best, R B; Vattulainen, I; M
onticelli, L; Periole, X; Tieleman, D P; de Vries, A H; Marrink, S J;  Nature Me
thods 2021; 10.1038/s41592-021-01098-3
    INFO - general - A classical Martini is made with up to 2 sizes of olives, a
lthough newer variants can contain up to three sizes of olives. -- Peter C Kroon
(base) rvce-bt-06@rvcebt06-HP-280-G3-MT:~/Desktop/purushotham_bbt21/protein_liga
nd_demo$
```

Martinize: Conversion of All atomic to Coarse grain model

4 Add water and ions using Insane.py script

python2 insane.py -f 1m4i_CG.pdb -o 1m4i_CG.gro -pbc cubic -box 10,10,10 -salt 0.15 -charge auto -sol W

copy the number of water and ions (without signs) to Topology file (1m4i_ONLY.top), also add required itp files and rename it to 1m4i.top

```
(base) rvce-bt-06@rvcebt06-HP-280-G3-MT:~/Desktop/purushotham_bbt21/protein_liga
nd_demo$ python2 insane.py -f 1m4i_CG.pdb -o 1m4i_CG.gro -pbc cubic -box 10,10,1
0 -salt 0.15 -charge auto -sol W
; NDX Solute 1 427
; Charge of protein: -3.000000
; NDX Membrane 428 0
; Charge of membrane: 0.000000
; Total charge: -3.000000
; NDX Solvent 428 9174
; NDX System 1 9174
; "I mean, the good stuff is just INSANE" --Julia Ormond
W             8558
NA+             96
CL-             93
(base) rvce-bt-06@rvcebt06-HP-280-G3-MT:~/Desktop/purushotham_bbt21/protein_liga
nd_demo$
```

Solvation and Ionisation

5 Insert Ligand molecule

Ligand has been parameterised and included for simulation

gmx insert-molecules -f 1m4i_CG.gro -nmol 1 -ci KAN.gro -o 1m4i_KAN.gro -replace

replace water by pressing the water selection. After successfull execution, change the number of water molecules and add kanamycin in molecules section and itp file of kanamycin in the topology file.

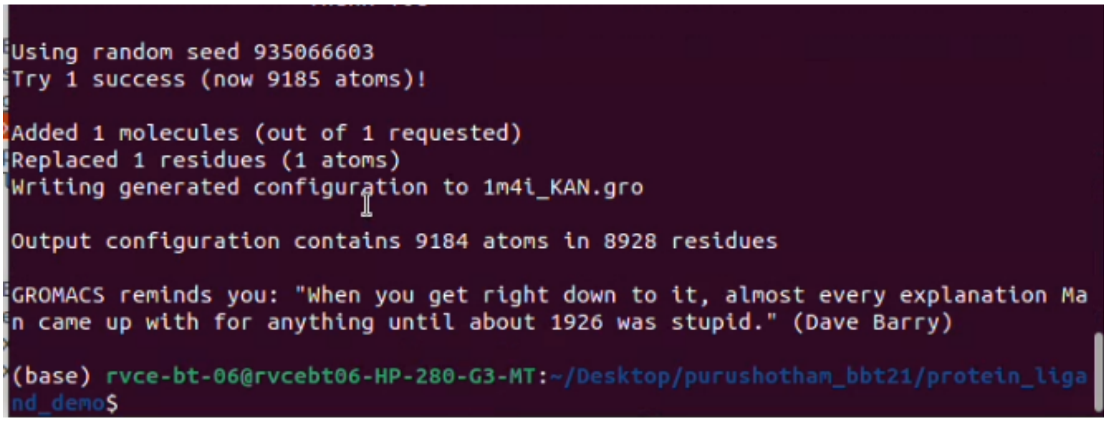

Addition of small molecule

6 Energy Minimization

gmx grompp -p 1m4i_KAN.top -f min.mdp -c 1m4i_KAN.gro -r 1m4i_KAN.gro -o em.tpr -maxwarn 1

gmx mdrun -v -deffnm em

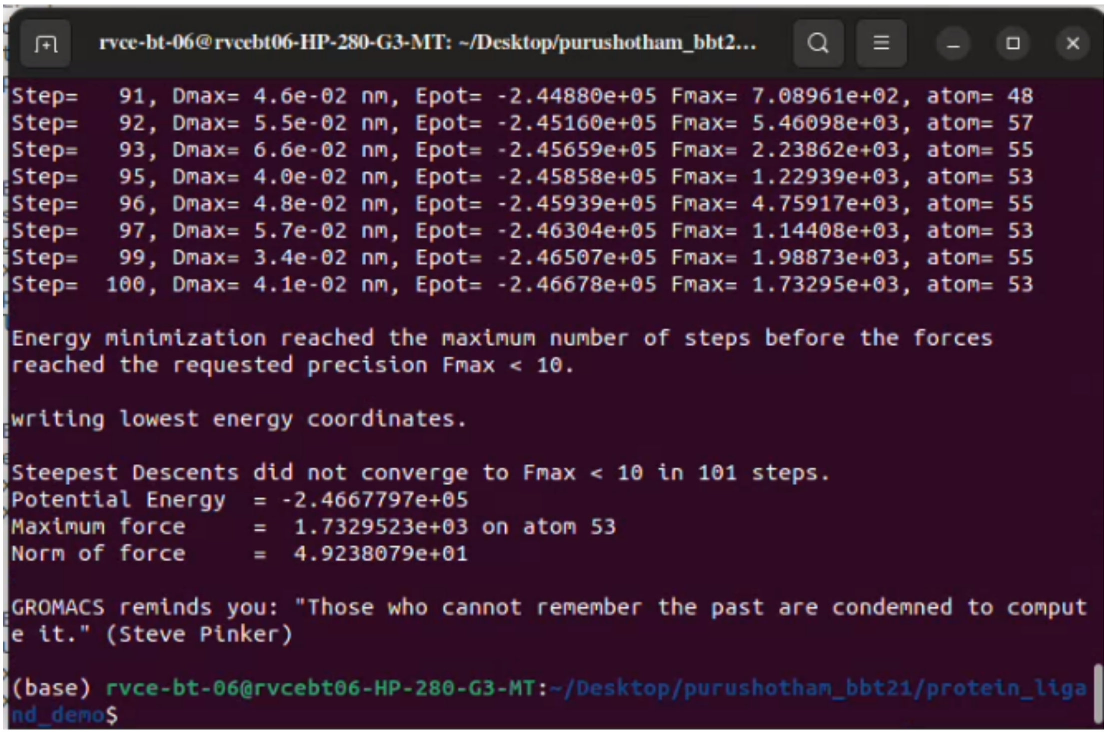

Energy Minimization

7 Equilibration

gmx grompp -p 1m4i_KAN.top -f eq.mdp -c em.gro -r 1m4i_KAN.gro -o eq.tpr -maxwarn 1

gmx mdrun -v -deffnm eq

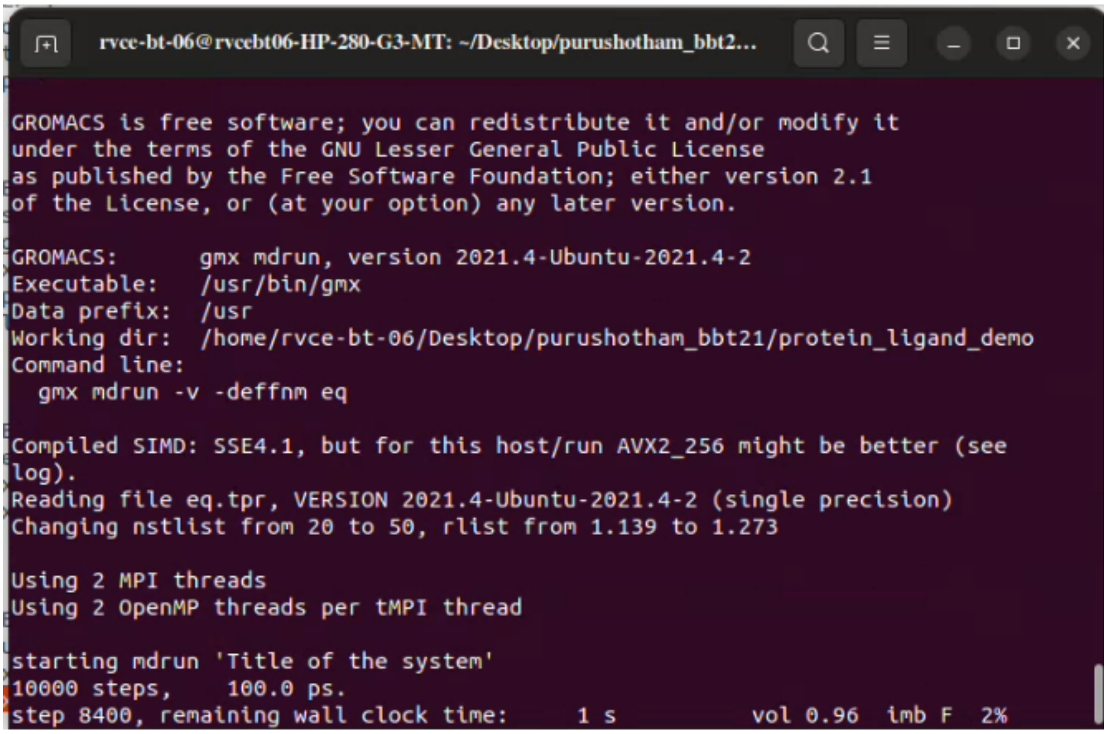

```
rvce-bt-06@rvcebt06-HP-280-G3-MT: ~/Desktop/purushotham_bbt2...

GROMACS is free software; you can redistribute it and/or modify it
under the terms of the GNU Lesser General Public License
as published by the Free Software Foundation; either version 2.1
of the License, or (at your option) any later version.

GROMACS:      gmx mdrun, version 2021.4-Ubuntu-2021.4-2
Executable:   /usr/bin/gmx
Data prefix:  /usr
Working dir:  /home/rvce-bt-06/Desktop/purushotham_bbt21/protein_ligand_demo
Command line:
  gmx mdrun -v -deffnm eq

Compiled SIMD: SSE4.1, but for this host/run AVX2_256 might be better (see
log).
Reading file eq.tpr, VERSION 2021.4-Ubuntu-2021.4-2 (single precision)
Changing nstlist from 20 to 50, rlist from 1.139 to 1.273

Using 2 MPI threads
Using 2 OpenMP threads per tMPI thread

starting mdrun 'Title of the system'
10000 steps,    100.0 ps.
step 8400, remaining wall clock time:     1 s          vol 0.96  imb F  2%
```

Equilibration

8 MD Production

I have ran for 2ns only to show, but you should increase it to case study.

gmx grompp -p 1m4i_KAN.top -f md.mdp -c eq.gro -o md.tpr

gmx mdrun -v -deffnm md

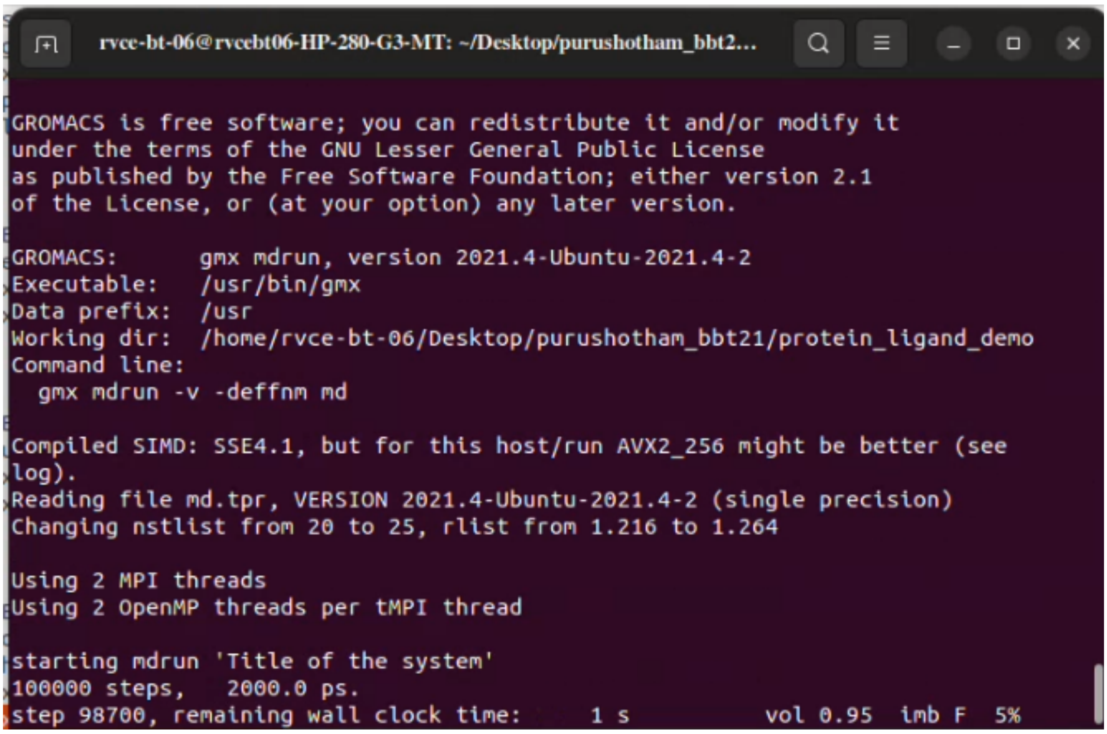

```
rvce-bt-06@rvcebt06-HP-280-G3-MT: ~/Desktop/purushotham_bbt2...

GROMACS is free software; you can redistribute it and/or modify it
under the terms of the GNU Lesser General Public License
as published by the Free Software Foundation; either version 2.1
of the License, or (at your option) any later version.

GROMACS:      gmx mdrun, version 2021.4-Ubuntu-2021.4-2
Executable:   /usr/bin/gmx
Data prefix:  /usr
Working dir:  /home/rvce-bt-06/Desktop/purushotham_bbt21/protein_ligand_demo
Command line:
  gmx mdrun -v -deffnm md

Compiled SIMD: SSE4.1, but for this host/run AVX2_256 might be better (see
log).
Reading file md.tpr, VERSION 2021.4-Ubuntu-2021.4-2 (single precision)
Changing nstlist from 20 to 25, rlist from 1.216 to 1.264

Using 2 MPI threads
Using 2 OpenMP threads per tMPI thread

starting mdrun 'Title of the system'
100000 steps,   2000.0 ps.
step 98700, remaining wall clock time:     1 s          vol 0.95  imb F  5%
```

MD Production
